# Supplementary figures and images for: Insights from circulating microRNAs in cardiovascular entities in turner syndrome patients
Source: PLoS One. 2020 Apr 9;15(4):e0231402. doi: 10.1371/journal.pone.0231402 (PMC7145016; doi:10.1371/journal.pone.0231402)

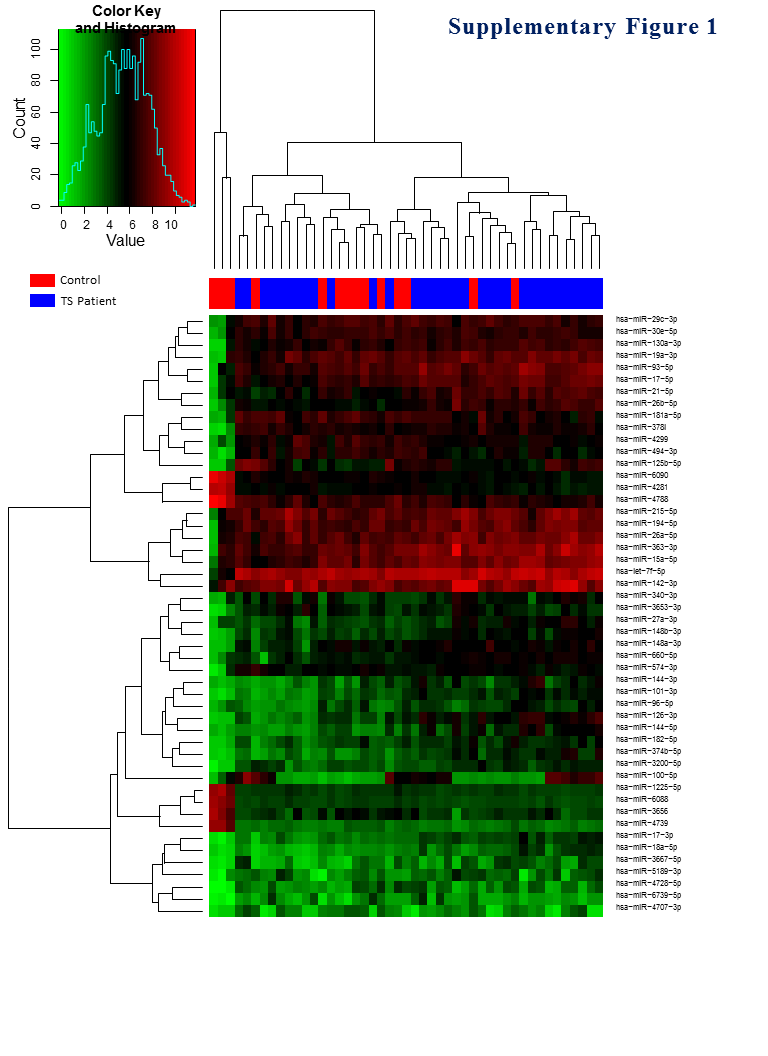

Supplement: S1 Fig — (TIF) [file pone.0231402.s001.tif]
